# New Route of Importation of *Mycobacterium tuberculosis* Beijing Genotype

**DOI:** 10.3201/eid1201.041214

**Published:** 2006-01

**Authors:** Darío García de Viedma, Fernando Chaves, Jesús Iñigo, for the Tuberculosis Molecular Epidemiology Study Group

**Affiliations:** * Hospital Gregorio Marañón, Madrid, Spain;; † Hospital Doce de Octubre, Madrid, Spain;; ‡ Consejería de Sanidad, Madrid, Spain

**Keywords:** molecular epidemiology, tuberculosis molecular epidemiology, Beijing, letter

**To the Editor:** *Mycobacterium tuberculosis* (MTB) Beijing genotype is spread throughout the world, and the highest prevalence has been detected in Asia and Eurasia. In western Europe, the prevalence of Beijing strains is relatively low (*1**–**3*), and cases in immigrants with Beijing strains are mainly Asian in origin.

In Spain, the last few years have been a key period for infectious diseases because of the sharp increase in the immigrant population. The percentage of tuberculosis (TB) cases in immigrants in our area has increased from 6.7% (75/1,123) in 1997–1999 to 29.4% in 2002–2003 (184/625). Our study had 2 aims: 1) to determine the prevalence of Beijing strains in Madrid and thus establish a reference for this genetic family before the impact of nonnative TB cases is experienced, and 2) to check whether the general parameters found for Beijing strains in Europe are valid for Spain or whether specific features can be identified.

All the MTB isolates (n = 510; ≈30% of all MTB cases in Madrid), obtained from patients January 2001–December 2002 from 2 tertiary teaching hospitals in Madrid (population 1,383,790), were genotyped by IS*6110* restriction fragment length polymorphism (RFLP) and spoligotyping. The distribution of nationalities of the patients from the studied population did not differ from the distribution in Madrid as a whole; therefore, no selection bias is expected.

Beijing strains were identified in 8 case-patients (1.6%). Spoligotypes corresponded to the Beijing standard patterns (Figure A). The RFLP types showed >13 bands (Figure B). All isolates were susceptible to anti-TB drugs (except 1 streptomycin-resistant strain).

**Figure:**
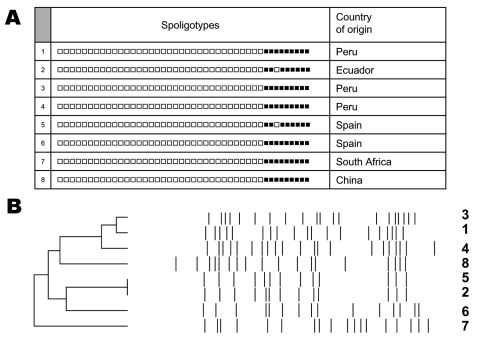
A) Spoligotypes from *Mycobacterium tuberculosis* (MTB) clinical isolates. Black boxes indicate hybridization with the corresponding spacer in the directed repeats region, and white boxes indicate the absence of hybridization. The country of origin and identification number for each patient are indicated. B) Similarity dendrogram of restriction fragment length polymorphism types obtained with MTB clinical isolates. Numbers on the right correspond to patient identification numbers.

Six of 8 patients infected with Beijing strains were immigrants; 4 were from South America (3 from Peru, with no epidemiologic links between them, and 1 from Ecuador), 1 was from South Africa, and 1 was from Asia. The remaining 2 patients were indigenous to Spain. Patients from Latin America had an increased prevalence of the Beijing genotype (3.8%) compared to the Spanish patients (0.4%).

Although Asia has been the source of importation of Beijing strains in the Netherlands, Denmark, and Italy (*1**–**3*), our data showed a potential new South American source for imported Beijing strains in Europe. Most immigrants in Madrid come from South America (and make up 71.8% of the total immigrant population, excluding western Europeans), and 59.3% of all South Americans come from Peru and Ecuador. Therefore, this new route of importation will likely have an impact. A previous report from Spain (*4*) described the isolation of Beijing strains from Peruvian patients, which suggests that this finding is not recent or casual. Few studies have offered genotyping data of Beijing strains in Peru and Ecuador.

Despite the low prevalence of Beijing strains in our area, we found 2 patients infected by the same strain (Figure), which suggests transmission between them. Notably, this recent transmission event involved a case in an immigrant patient (Ecuadorian, patient 2) and an indigenous patient (patient 5) (Figure). Specimens from the Ecuadorian patient were positive for acid-fast bacilli; his TB symptoms appeared 2 months before his arrival in Spain, and he began anti-TB therapy 7 months after arrival. Studies focused on the analysis of recent transmission of strains between those born in other countries and the autochthonous population in Europe generally conclude that the transfer of strains between both groups is low or moderate (*5**–**7*). In Madrid, recent transmission between the immigrant and autochthonous population has been frequently detected (*8*), likely indicating a fairly high degree of social permeability between the 2 groups. The transmission of Beijing MTB from a person born in another country to a person born in Spain reported here seems to follow this trend and raises concern about a potential spread of Beijing MTB to the autochthonous population.

The isolate from the other Spanish patient (patient 6) shared the genotype of the strain described earlier in the spread of Beijing genotype in Gran Canaria (*9*); a Liberian was the first case-patient. The patient from Spain in our report had been imprisoned on that island before her arrival in Madrid. These data suggest another way Beijing genotype can be imported into Spain, which is different from the South American route.

In summary, we describe TB patients with Beijing genotype strains in Madrid among patients from South America. This geographic origin differs from the predominant Asian origin reported for TB cases in other European countries caused by the Beijing genotype. Our findings suggest an alternate route of transmission between South America and Europe for the Beijing genotype. Furthermore, the recent occurrence of this genotype in a TB patient from Spain, who shared an RFLP type with a South American patient, suggests further transmission of these strains into the local community. Longitudinal studies should monitor the potential impact and establishment of these strains after their introduction.
